# A Model of the 2014 Ebola Epidemic in West Africa with Contact Tracing

**DOI:** 10.1371/currents.outbreaks.846b2a31ef37018b7d1126a9c8adf22a

**Published:** 2015-01-30

**Authors:** Glenn Webb, Cameron Browne, Xi Huo, Ousmane Seydi, Moussa Seydi, Pierre Magal

**Affiliations:** Department of Mathematics, Vanderbilt University, Nashville, Tennessee, USA; Department of Mathematics, Vanderbilt University, Nashville, Tennessee, USA; Department of Mathematics, Ryerson University, Toronto, Ontario, Canada; Ecole Polytechnique de Thiès, Thiès, Sénégal; Department of Infectious Diseases, Cheikh Anta Diop University, Dakar, Senegal; Université de Bordeaux, Bordeaux, France

**Keywords:** ebola

## Abstract

A differential equations model is developed for the 2014 Ebola epidemics in Sierra Leone and Liberia. The model describes the dynamic interactions of the susceptible and infected populations of these countries. The model incorporates the principle features of contact tracing, namely, the number of contacts per identified infectious case, the likelihood that a traced contact is infectious, and the efficiency of the contact tracing process. The model is first fitted to current cumulative reported case data in each country. The data fitted simulations are then projected forward in time, with varying parameter regimes corresponding to contact tracing efficiencies. These projections quantify the importance of the identification, isolation, and contact tracing processes for containment of the epidemics.

## Introduction

Our objective is to develop a mathematical model of the 2014 Ebola epidemic in West Africa. The model consists of a system of ordinary differential equations for the compartments of the epidemic population. The model incorporates the unique features of the Ebola outbreaks in this region. These features include the rates of transmission to susceptibles from both infectious cases and improperly handled deceased cases, the rates of reporting and isolating these cases, and the rates of recovery and mortality for these cases. The model also incorporates contact tracing^2^ of reported infectious cases, and analyzes the efficiency of the identification and isolation of these cases, and the efficiency of contact tracing measures.

We apply the model to Sierra Leone and Liberia, first fitting WHO data for each country from outbreak in the spring of 2014 to September 23, 2014. We then simulate forward projections of the epidemic in each of these countries, based on varied efficiencies in identifying, isolating, and contact tracing of infected individuals. Our model predictions indicate that the containment of the epidemic requires a high level of both the general identification and isolation process and the contact tracing process for removing infectious individuals from the susceptible population.

## The Model

**Figure:**
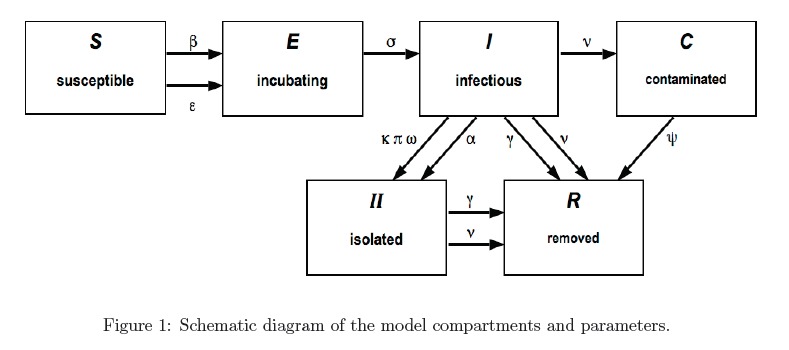

The model, which is of SEIR form^1^^,^ 3 ^,^ 7 ^,^ 8 ^,^ 9, incorporates specific features of contact tracing in the current epidemics. The model consists of the populations at time *t *of susceptibles S(t) (capable of becoming infected), exposed E(t) (incubating infected), I(t) (infectious infected), contaminated deceased C(t) (improperly handled corpses of infected), isolated infectious II(t) (exposed and infectious infected who have been identified and isolated from the susceptible population), and removed R(t) (infectious cases who have recovered or died). The compartments II(t) and R(t) de-couple from the other compartments, and their values can be obtained from S(t), E(t), I(t), C(t). A schematic diagram of the model is shown in figure 1. The system of differential equations for S(t), E(t), I(t), C(t) is

\begin{equation*}S'(t)=- \beta S(t)\frac{I(t)}{N}- \epsilon S(t) \frac{C(t)}{N}\end{equation*}

\begin{equation*}\%0AE\%27(t)= \beta S(t)\frac{I(t)}{N}+  \epsilon S(t) \frac{C(t)}{N}- \sigma E(t)\%0A\end{equation*} \begin{equation*}I\%27(t)= \sigma E(t)- \alpha I(t) - \gamma I(t)-  \nu I(t)- \kappa\pi\omega (\alpha I(t)+\psi C(t)) \end{equation*}

\begin{equation*}C'(t)=\nu I(t) - \psi C(t)\end{equation*}

We note that the transmission terms in the S(t) equation are of mass-action form. We assume that the initial conditions and parameters are such that I(t) remains non-negative in a reasonable time-frame. The parameters of the model are given in Table 1.

A major goal of our study is to fit the model to current reported data for Sierra Leone, Liberia, and Guinea. We note that the data available are the cumulative clinical reported cases^15^^,^ 16, that is, the suspected cases, probable cases and confirmed cases according to the definitions given in WHO^17^. Therefore, if we denote by CUM(t) the cumulative reported cases at time \begin{equation*}\small{t}\end{equation*}, then at time \begin{equation*}\small{t+\Delta t}\end{equation*}we have:

\begin{equation*}CUM(t+\Delta t)= CUM(t)+ \int_t^{t+\Delta t}\alpha I(s) \,ds + \int_t^{t+\Delta t} \psi C(s) \,ds \end{equation*}

For our model it can be proved that as \begin{equation*}\small{t\rightarrow\infty}\end{equation*} the cumulative fraction of reported cases \begin{equation*}\small{CUM(t) / (N-S(t))}\end{equation*} converges to \begin{equation*}\small{(\alpha + \nu) / (\alpha + \nu + \gamma)}\end{equation*}. Further, after a brief initial time period, the cumulative fraction of reported cases in a gradually increasing function of time with two plateaus mirrored and reflected about the time at which I(t) is maximum (see Figure 3). In general, it is difficult to identify a time-independent probability that a case will be reported, since data are not known for unreported cases. The issue of unreported cases is very important for contact tracing. A major goal of contact tracing is to remove from the epidemic population a sufficient number of infectious cases to contain the epidemic, but only reported cases are contact traced.

## Parameters in the Model

**Figure:**
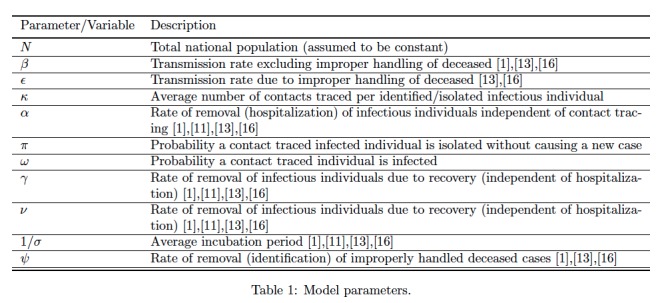

The parameters of the model are given in Table 1. The values of the parameters \begin{equation*}\small{ \ \beta,  \ \epsilon, \ \psi} \end{equation*} and \begin{equation*}\small{\alpha}\end{equation*} are estimated for the three countries using a least square curve fitting algorithm. In addition, the initial values S(0), E(0), I(0) and C(0) are also estimated using the least squares algorithm. There are many different parameter combinations which fit, thus confidence intervals may not be relevant for our purposes. The goal is to obtain a reasonable visual fit, and then to illustrate general qualitative features of the impact of contact tracing on the epidemic trajectory, along with identifying rough rules of thumb for containing the epidemic based on numerical simulations.

The parameters \begin{equation*}\small{\sigma}\end{equation*} and \begin{equation*}\small{\nu}\end{equation*} are taken to be values suggested by references in Table 1. The parameter \begin{equation*}\small{\gamma}\end{equation*} is assumed to be a value such that the case mortality rate (outside hospital) is approximately 80%. In fitting the model to the data, we assume that contact tracing does not occur (\begin{equation*}\small{\kappa=0}\end{equation*}) during the time period of the data (up until September 23, 2014). The reasons for this assumption are that contact tracing has been insufficient in the three countries, and to reduce the number of parameters to estimate in fitting the data. As stated before, the main goal for incorporating contact tracing is to project forward the impact of effective contact tracing on the future number of cases. The basic reproduction number of the model (1) is given by the following formula: (computed by the next generation method^13^):

\begin{equation*}\mathcal R_0=\frac{\beta}{(\alpha+\nu)(1+\kappa\pi\omega)+\gamma}+\frac{\nu\epsilon}{\psi((\alpha+\nu)(1+\kappa\pi\omega)+\gamma)}\end{equation*}

The \begin{equation*}\small{\mathcal R_0}\end{equation*} values we obtain are similar to the \begin{equation*}\small{\mathcal R_0}\end{equation*} values obtained in^1^^,^ 3 ^,^ 4 ^,^ 5 ^,^ 12 ^,^ 18.

The removal rate of infectious individuals due to contact tracing at time \begin{equation*}\small{t}\end{equation*} has the following form:


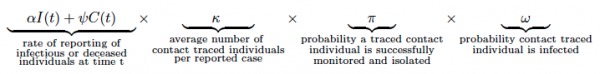


The probabilities \begin{equation*}\small{\omega}\end{equation*} and \begin{equation*}\small{\pi}\end{equation*} can, in principle, be ascertained from records of contact traced cases over an interval of time. We note that the probability \begin{equation*}\small{\omega}\end{equation*} that a traced contact is infected will depend on the average number of traced contacts \begin{equation*}\small{\kappa}\end{equation*}. The values of \begin{equation*}\small{\omega}\end{equation*}, \begin{equation*}\small{\kappa}\end{equation*}, and \begin{equation*}\small{\pi}\end{equation*} can be updated as health authorities collect data of hospitalized and deceased cases. The parameter \begin{equation*}\small{\pi}\end{equation*} measures the efficiency of the tracking, monitoring, and removal of contact traced infectious cases for a specific organization and implementation of a contact tracing process. In particular, \begin{equation*}\small{\pi}\end{equation*} measures how efficiently public health workers remove infected individuals upon symptoms onset, and prevent secondary transmissions. The solution of the model projects the likelihood that an implemented contact tracing process will contain the epidemic, and if not, then the contact tracing process must be enhanced.

The entire contact tracing process is highly dependent on public health resources, and varies greatly in different locations and epidemic stages. For example, in Sénégal the following policy has been implemented:

1. each identified patient is questioned in order to obtain a complete list of contacts;
2. the contacts are traced;
3. each contact is asked to stay at home;
4. each day, for 21 days, a healthcare worker visits the contacts and verifies whether or not the contacts are showing symptoms.

These protocols are rigorous and have been successful in preventing new cases in Sénégal. On October 17, 2014, the World Health Organization declared the end of the outbreak of the Ebola epidemic in Sénégal (after 42 days with no new cases and with active surveillance demonstrably in place and supported by good diagnostic capacity)^19^ .

## Simulations of the Ebola Epidemic in Sierra Leone

In Figure 2 we fit the model without contact tracing to the cumulative reported case data for Sierra Leone from May 27, 2014 to September 23, 2014 (WHO^15^^,^ 16). The fit to data can be accomplished with varying combinations of parameters. Here we have used a least-squares algorithm to obtain a choice of parameters with relatively accurate fit. The parameters obtained in the fit yield a basic reproduction number of \begin{equation*}\small{\mathcal R_0=1.26}\end{equation*}. The simulation yields the following information about the epidemic on September 23, 2014 (day 119): the ratio of exposed cases to infectious cases is \begin{equation*}\small{E(119) / I(119) \approx 2.49}\end{equation*}; the ratio of improperly handled deceased cases to infectious cases is \begin{equation*}\small{C(119) / I(119) \approx 0.57}\end{equation*}; and the ratio of cumulative reported cases to cumulative unreported cases is \begin{equation*}\small{\approx 1.78}\end{equation*}. These ratios, which are dependent on parameters, are relatively stable at the data end-stage. In Figure 3 we graph the model simulation of the projected fraction of cumulative reported cases as a function of time without contact tracing. The fraction of cumulative reported cases to total cases shows a two phase behavior with a lower value transitioning to an upper value. In Figure 4 we add contact tracing to the model and predict the further evolution of the epidemic in Sierra Leone forward from September 23, 2014. The contact tracing parameters \begin{equation*}\small{\alpha}\end{equation*} and \begin{equation*}\small{\kappa}\end{equation*} are varied in a sensitivity analysis, while the other contact tracing parameters are held constant. The graphics reveal that a general identification/isolation rate \begin{equation*}\small{\alpha>0.3}\end{equation*} is required for containing the epidemic. The number \begin{equation*}\small{\kappa}\end{equation*} of contacts traced per identified case is also important if \begin{equation*}\small{\alpha}\end{equation*} is smaller. After contact tracing begins and for a short time, the reported cases increase as \begin{equation*}\small{\kappa}\end{equation*} increases, but then the epidemic subsides as contact tracing takes effect.

**Figure:**
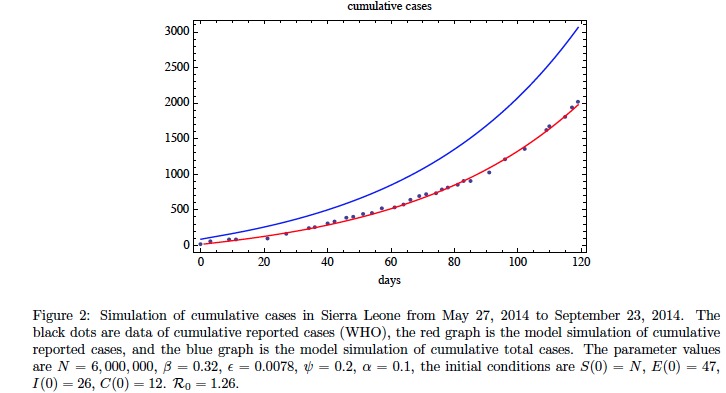

**Figure:**
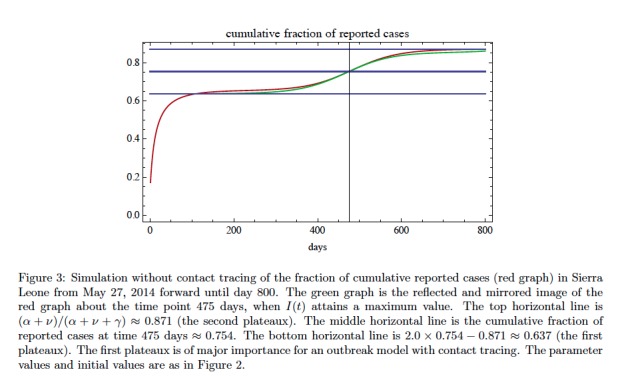

**Figure:**
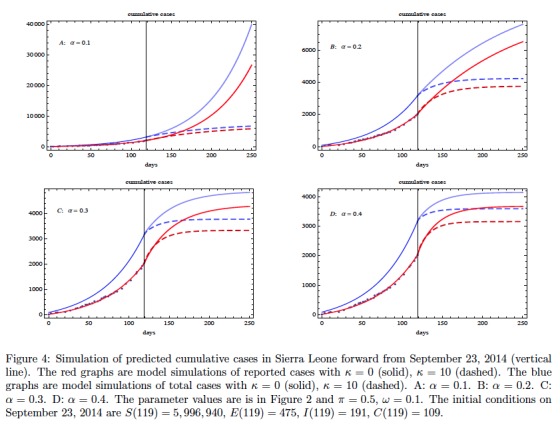

## Simulations of the Ebola Epidemic in Liberia

In Figure 5 we fit the model without contact tracing to the cumulative reported case data for Liberia from June 17, 2014 to September 23, 2014 (WHO^15^^,^ 16). We have used a least-squares algorithm to obtain a choice of parameters with relatively good fit (other parameter choices will give similar fits). The parameters obtained in the fit yield a basic reproduction number of \begin{equation*}\small{\mathcal R_0=1.54}\end{equation*}. The simulation yields the following information about the epidemic on September 23, 2014 (day 98): the ratio of exposed cases to infectious cases is \begin{equation*}\small{E(98) / I(98) \approx 3.35}\end{equation*}; the ratio of improperly handled deceased cases to infectious cases is \begin{equation*}\small{C(98) / I(98) \approx 0.58}\end{equation*}; and the ratio of cumulative reported cases to cumulative unreported cases is \begin{equation*}\small{ \approx 1.37}\end{equation*}. These ratios, again depend on parameters, are very stable throughout most of the period of simulation. In Figure 6 we add contact tracing to the model and predict the further evolution of the epidemic in Liberia forward from September 23, 2014. The contact tracing parameters \begin{equation*}\small{\alpha}\end{equation*} and \begin{equation*}\small{\pi}\end{equation*} are varied in a sensitivity analysis, while all the other contact tracing parameters are held constant. The graphics again reveal that an identification/isolation rate \begin{equation*}\small{\alpha > 0.3}\end{equation*} is required for containing the epidemic. The role of \begin{equation*}\small{\pi}\end{equation*}, as the probability of efficiently tracing and monitoring a contact traced individual is also important for the containment of the epidemic. As in Figure 4, the reported cases increase for a short time as \begin{equation*}\small{\pi} \end{equation*} increases, but then decrease as contact tracing takes effect.

We could not obtain data for the parameters \begin{equation*}\small{\omega}\end{equation*} and \begin{equation*}\small{\pi}\end{equation*}. In principle, \begin{equation*}\small{\omega}\end{equation*} should depend on the probability of transmission, and some efficiency in the breadth and accuracy of contact tracing. Since, we are not sure of the parameter, we choose an arbitrary probability value of \begin{equation*}\small{\omega=0.1}\end{equation*} which seems reasonable. As \begin{equation*}\small{\omega}\end{equation*} increases (decreases), contact tracing will have a larger (smaller) impact on the epidemic trajectory. Also, \begin{equation*}\small{\pi}\end{equation*} is a control parameter describing efficiency of monitoring and removal (if infectious) of traced contacts. We vary this parameter in Fig. 6, to show qualitative effects of varying \begin{equation*}\small{\pi}\end{equation*} to specific values (\begin{equation*}\small{\pi=0.4,\ 0.6}\end{equation*}). Note that the effect of varying \begin{equation*}\small{\omega}\end{equation*}and \begin{equation*}\small{\pi}\end{equation*} on the dynamics are identical since the terms always appear as a product in the differential equation. Based on numerical simulations, we conclude that if \begin{equation*}\small{\alpha>0.3}\end{equation*} and \begin{equation*}\small{\pi>0.5}\end{equation*}, then the epidemic can be contained after a short window of time.

**Figure:**
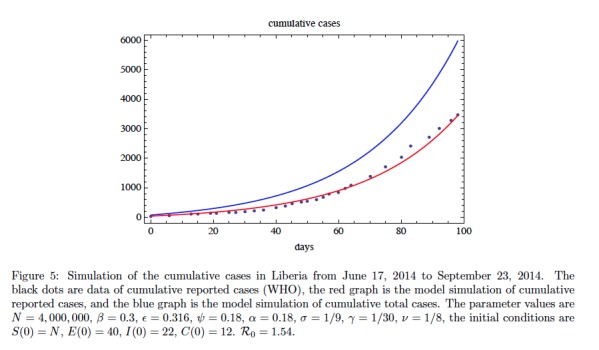

**Figure:**
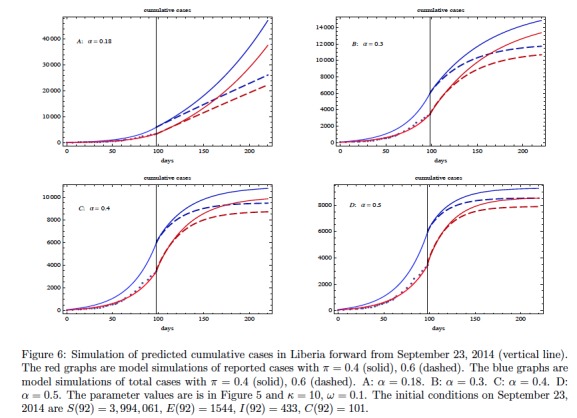

## A Stochastic Version of the Model

Figure 7 shows 100 stochastic simulations compared with the ODE solution for contact tracing in Sierra Leone with different rates of case hospitalization \begin{equation*}\small{\alpha}\end{equation*} and average number of contacts traced \begin{equation*}\small{\kappa}\end{equation*}. The stochastic simulations are generated by simulating a Continuous Time Markov Chain as a continuation of the ODE solution beginning at the last data time point. All other parameters are the same as in Figure 4. The averages of the stochastic model solutions agree with the ODE solutions of Model 1.

**Figure:**
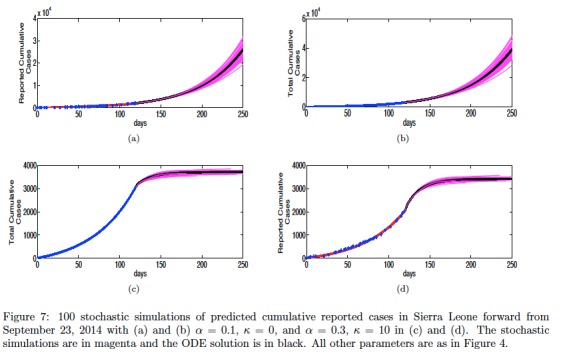

## Summary and Conclusions

We have developed a model of Ebola epidemics in West Africa that focuses attention on the elements of public health policies for containment of these epidemics. Our simulations for Sierra Leone, Liberia, and Guinea fit the cumulative reported cases for these countries up to September 23, 2014, and project future epidemic levels forward from September 23, 2014 (based on various parameterizations corresponding to these elements). Our projections indicate that the most important elements for containment of the epidemics within a relatively short time span are that

1. infectious cases (independent of contact tracing) are efficiently reported and isolated, with the average time between the appearance of symptoms and isolation less than 3 days (\begin{equation*}\small{\alpha > 0.3}\end{equation*});
2. contact traced incubating infected cases are efficiently monitored, with average probability of compliance, with isolation upon appearance of symptoms (such that no new cases are caused by individual), greater than 0.5 (\begin{equation*}\small{\pi > 0.5}\end{equation*}).

Also of importance in mitigation of the epidemics is a reduced rate at which infected deceased are improperly handled (\begin{equation*}\small{\psi, \ \  \nu}\end{equation*}), a sufficient number of contacts traced per identified infectious individual (\begin{equation*}\small{\kappa}\end{equation*}). The model allows quantification of the parameters corresponding to public health controls (\begin{equation*}\small{\alpha, \ \ \psi, \ \ \kappa, \ \ \pi, \ \ \ \omega}\end{equation*}) for evaluating the impact of public health policies for the evolution of these epidemics.
